# A rare case of congenital pupillary abnormality: a case report

**DOI:** 10.1186/s12886-022-02422-x

**Published:** 2022-05-02

**Authors:** Lancao Hao, Zicheng Ma, Chenjie Song, Siquan Zhu

**Affiliations:** Department of Ophthalmology, Beijing Anzhen Hospital, Capital Medical University, Beijing, 100029 China

**Keywords:** Pupillary abnormality, Microcoria, Pupilloplasty, Case report

## Abstract

**Background:**

Congenital anomalies of the pupil are quite varied, including abnormal size, shape, color, response to stimulus, and function. We are here reporting an unusual case presented with the absence of pupillary opening with folds of iris tissue at the center. Only an extremely small pupil (diameter < 0.5 mm) could be observed during the operation.

**Case presentation:**

A 15-year-old male patient visited our outpatient clinic due to vision difficulty in his right eye for more than ten years. The best-corrected visual acuity was 2.0 logMAR and 0 logMAR for the right and left eye, respectively. There were amblyopia, astigmatism and constant exotropia in his right eye. Ophthalmic examination of the right eye showed flat iris root, minimal iris pigmentation, and the pupil area was entirely covered by iris tissue. Lens status and fundus evaluation could not be commented. The left eye was found to be within normal limit. Based on ophthalmic examination, the admission diagnosis was given as acorea. Pupilloplasty was performed on the right eye due to the situation that the iris tissue blocked the visual axis, which led to visual impairment and stimulus deprivation amblyopia. However, an extremely small pupil at the center of his pupillary area was observed during the operation. The postoperative course was favorable, and a normal pupil was secured. Hospital discharge diagnosis was given as microcoria, and amblyopia treatment was followed.

**Conclusions:**

We report a rare case of congenital pupillary abnormality. The further diagnosis was given as microcoria, which should be differentiated from acorea. For this kind of pupil disorder which blocks the visual axis, early diagnosis and treatment can help prevent the development of stimulus deprivation amblyopia.

## Background

Congenital anomalies of the pupil are pretty varied, including anisocoria (pupils with unequal size), dyscoria (pupils with unusual shape), corectopia (pupils with abnormal position), leukocoria (white colored pupils), aniridia (pupils too large), microcoria (pupils too small), acorea (complete absence of pupils), persistent pupillary membrane (iris strands obscure the pupils) and paradoxical pupillary reaction [1].

Microcoria (MCOR), which also referred to as congenital miosis, is a rare autosomal dominant disease associated with structural variation of chromosome 13q31-q32 [2, 3]. It is characterized by a small pupil (diameter < 2 mm) results from the absent or underdeveloped dilator muscle fibers. Here we described a rare case of congenital pupillary abnormality. The abnormal clinical findings in our case were microcoria, amblyopia, astigmatism and exotropia.

## Case presentation

This case involved a 15-year-old male patient who presented to our outpatient department due to "vision difficulty in the right eye for more than ten years.” This patient had a noticeable pupillary abnormality in his right eye at birth which was found by his parents but wasn’t given treatment.

**Fig. 1:**
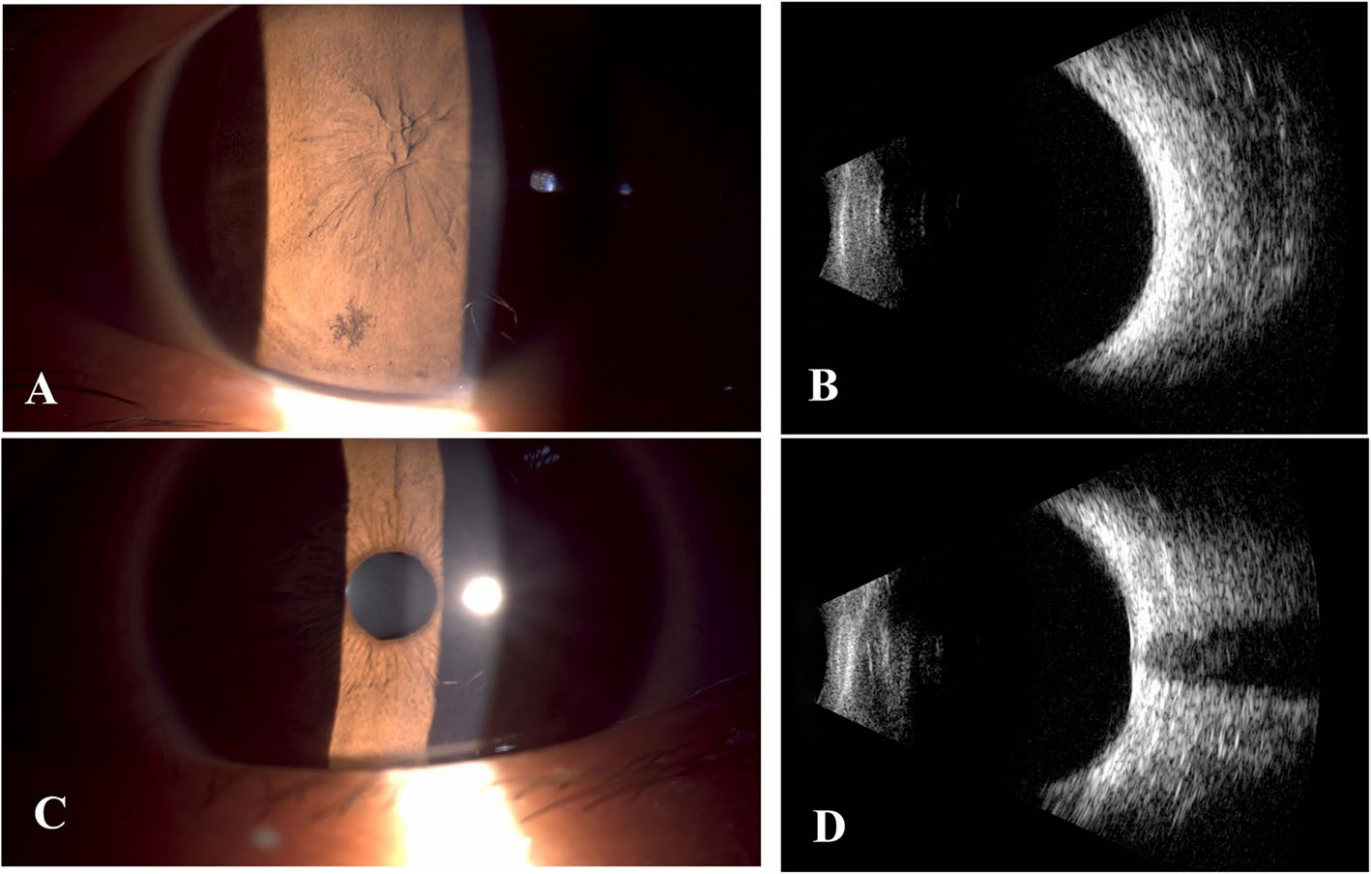
Preoperative examination of both eyes. **A** and **C** Preoperative slit-lamp examination for the right and left eye, respectively; **B** and **D** Preoperative B scan for the right and left eye, respectively

Ophthalmic examination revealed corneal astigmatism was 5.57 diopter (D) and 2.57 D respectively for the right and left eye, respectively. The best-corrected visual acuity (BCVA) was 2.0 logMAR and 0 logMAR for the right and left eye, respectively. A-Scan ultrasonography measured axial length of 23.33 mm and 23.50 mm, intraocular pressure of 14.7 mmHg and 9.1 mmHg, the number of corneal endothelial cells of 3427.2 /mm^2^ and 3156.7 /mm^2^ in his right and left eye, respectively. Slit-lamp examination of the right eye showed clear cornea, deep anterior chamber. The pupillary area is entirely covered by the iris tissue with the absence of pupillary opening, and iris hypoplasia with poor development of iris crypts and collarette. Gonioscopy revealed flat iris root, clear iridocorneal angle structures and minimal iris pigmentation. Lens status and fundus evaluation could not be commented. B-Scan ultrasound measurement was within normal limits. Constant exotropia in his right eye was observed, but extraocular muscle movements were normal. There was no other abnormality in the left eye (Fig. 1).

The patient was diagnosed with Tourette's syndrome in 2011. He had a full-term cesarean section. His mother had no history of fever, no exposure to radiation or chemical drugs during pregnancy. The parents were not consanguineous. Parents and other family members had no eye-related medical history or family genetic history.

**Fig. 2:**
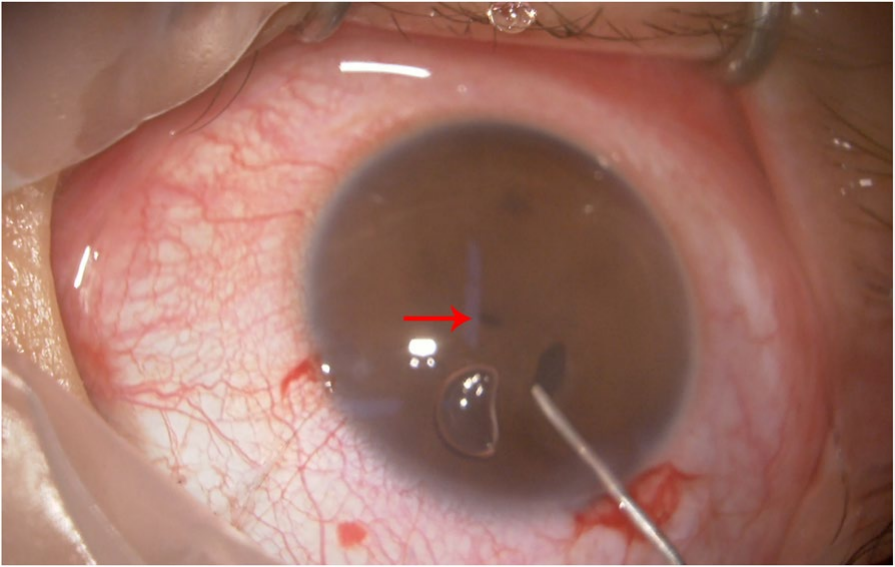
Intraoperative photograph of the right eye, note a small pupil (arrow)

Based on ophthalmic examination, the admission diagnosis was given as acorea. Pupilloplasty was performed on the right eye under topical anesthesia on July 29, 2021. Two superior clear corneal incisions were made using keratomes. After injecting a viscoelastic substance into the anterior chamber and posterior chamber, an extremely small pupil was observed in the pupil area at < 0.5 mm in diameter (Fig. 2). An iris scissor was moved along a circular path to cut off the iris tissue in the pupil area, and an auxiliary hook was used to help form a pupil of about 3 mm in diameter. During the operation, the lens was transparent without synechiae. The sterile balanced salt solution was used to hydrate the superior corneal wound. After the incision was closed, Tobramycin and Dexamethasone Ophthalmic Ointment was applied to the conjunctival sac, and the operative eye was covered after the operation.

**Fig. 3:**
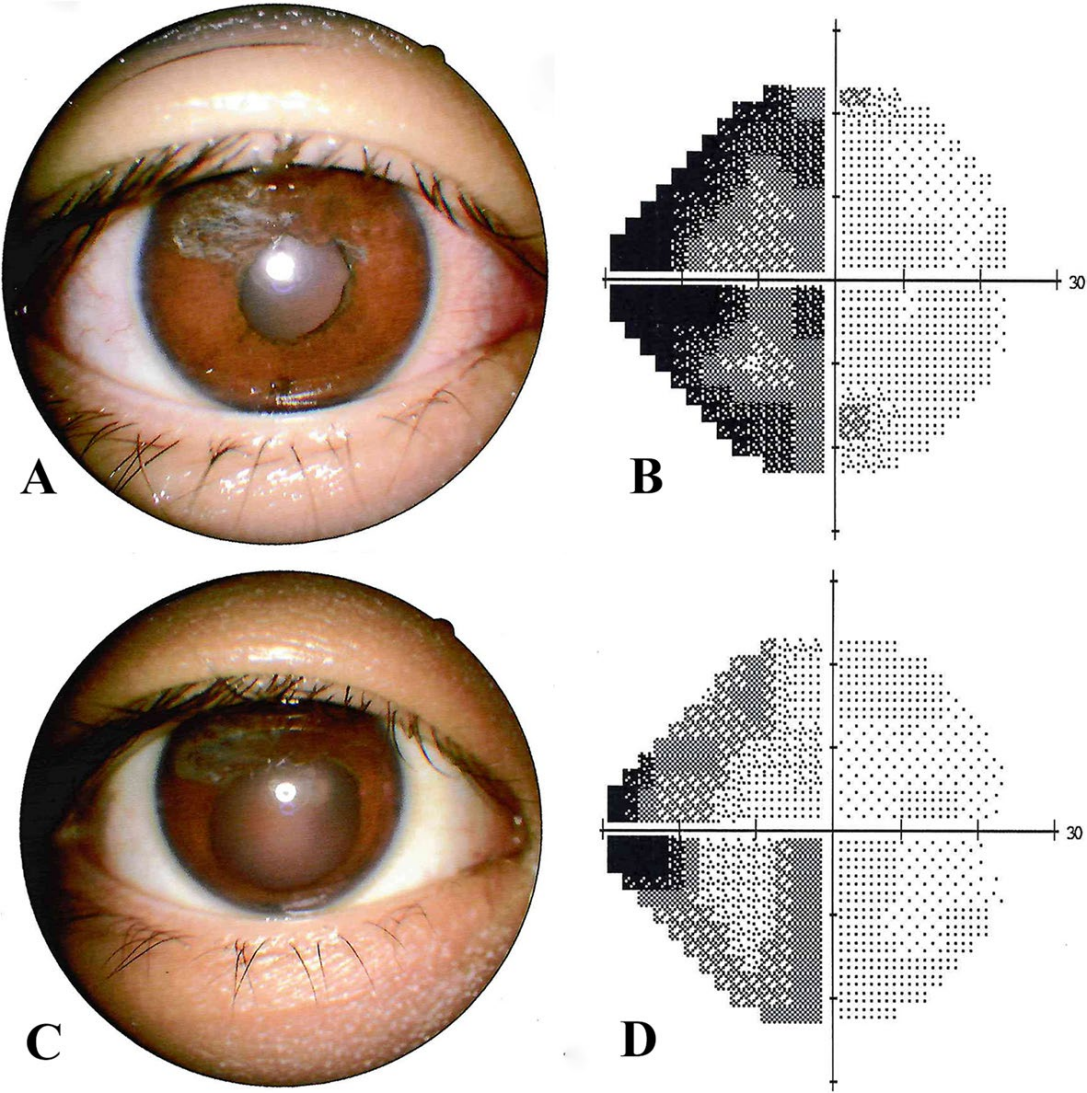
Postoperative mydriatic reaction and the visual field of the right eye. **A** and **C** The response to mydriatic drops after the surgery; **B** and **D** Changes of visual field before and after mydriasis

**Fig. 4:**
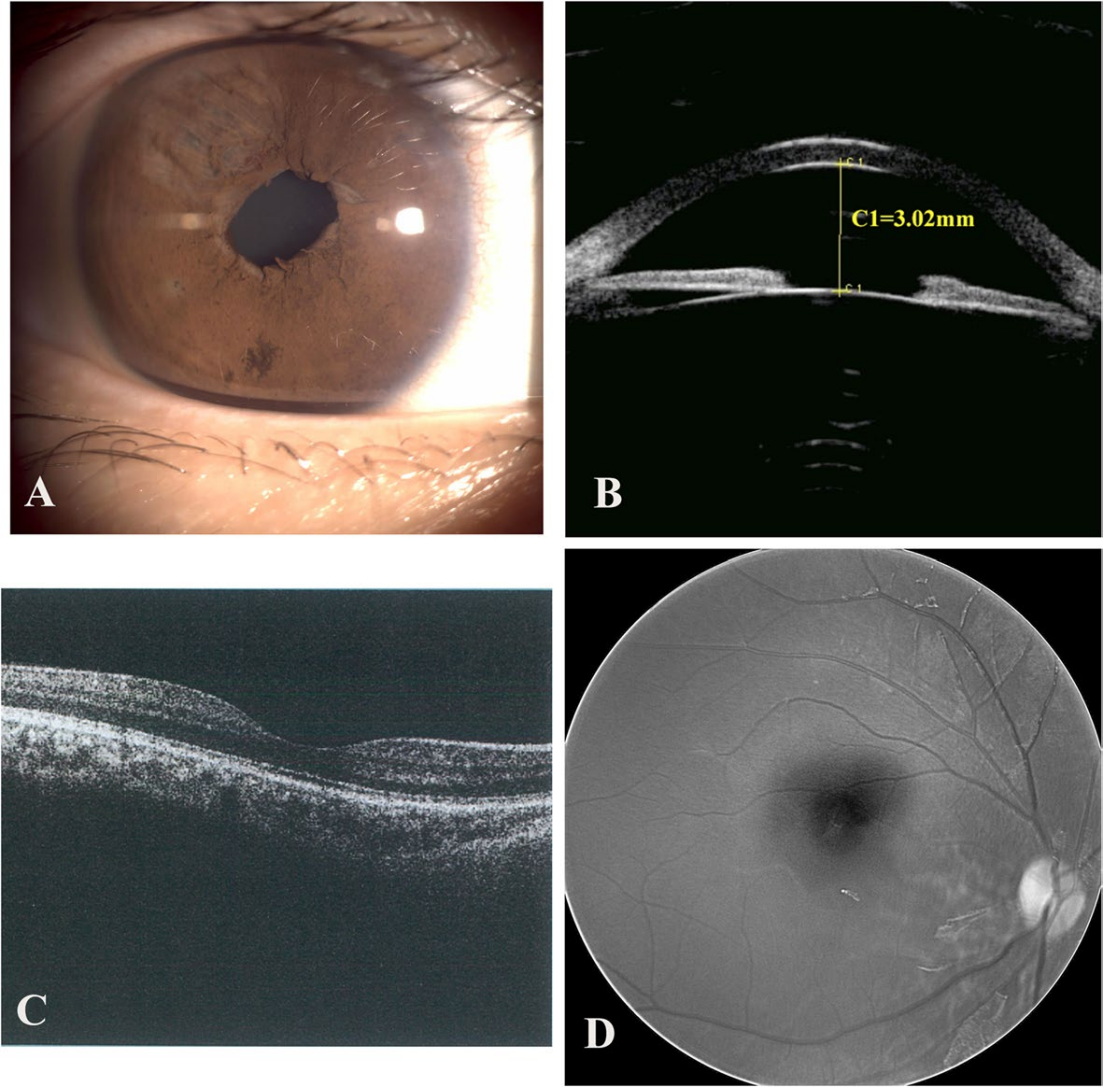
Postoperative examination of the right eye. **A** Central round pupil in the right eye after surgical treatment; **B** Postoperative UBM of the right eye; **C** Postoperative OCT of the right eye; **D** Postoperative fundus photography of the right eye

The postoperative course was favorable, and a normal pupillary area was secured. After the operation, the patient felt the vision was much brighter than before, but blurred slightly due to the loss of pinhole effect. Postoperative examination of the right eye showed that the BCVA was 2.0 logMAR not improving further, the number of corneal endothelial cells was 3375.9 /mm^2^, the anterior chamber was of normal depth, the pupil was round and central, the final pupil size measured on IOL Master was 4.1 mm. Pupil responded partially to topically administrated tropicamide eye drops. This dilatation was followed by visual field improvement (Fig. 3). Ultrasound biomicroscopy (UBM) showed clear iridocorneal angle structures. The lens and vitreous were transparent. Optical coherence tomography (OCT) and fundus photography revealed the macula was healthy and the architecture of the retinal layers appeared normal (Fig. 4). There were no postoperative surgical complications, hospital discharge diagnosis was given as microcoria, and amblyopia treatment was followed.

## Discussion and conclusions

The iris is composed of four layers: anterior border layer, stroma and sphincter muscle, anterior pigment epithelium layer and dilator muscle, and posterior pigment epithelium layer. Among them, the sphincter muscle can constrict the pupil in miosis and dilator muscle can expand the pupillary aperture in mydriasis to control the quantity of light to enter the eye [4, 5].

Congenital microcoria is an iris hypoplasia that affects the regulation of the amount of light reaching the retina. W. R. Wilde's essay of 1862 on " Malformations and Congenital Diseases of the Organ of Sight " is the first mention of the disease where he called it as “miosis congenita”. The definition of congenital microcoria was first described by Holth in 1923 [6]. Congenital microcoria manifested in pinhole pupil (< 2 mm), poor development of the crypt and collarette, thinning iris stroma with corresponding transillumination, abnormal iridocorneal angle with minimally pigmented [4, 7]. Congenital microcoria is associated with axial myopia, glaucoma, astigmatism, cataract and goniodysgenesis [8].

In our case, the patient had an extremely small pupil (diameter < 0.5 mm), iris hypoplasia with poor development of iris crypts and collarette, partial absence of pupil dilation, minimal iris pigmentation. Additional ocular anomalies were amblyopia, astigmatism and exotropia. The diagnosis was given as microcoria, which should be differentiated from acorea.

Acorea is a more severe anomaly of the pupil where the pupil is completely absent. It has been reported in human eyes for the first time in 2013 where they described a unique syndrome consisting of acorea, microphthalmia and cataract [9]. Ramasubramanian et al. [10] described a case whose slit‑lamp examination showed the absence of pupillary opening with folds of iris tissue at center, and uncorrected visual acuity was 3.0 logMAR with 35 prism diopter of exotropia. In our case, the preoperative slit-lamp examination of the right eye showed pupillary area is entirely covered by the iris tissue with the absence of pupillary opening, which led to preoperative diagnosis was given as acorea. But an extremely small pupil was observed during the operation, and we think the tiny hole in the pupillary area had a certain function, because the patient had pinhole effect before the operation, and binocular axis length were similar, which to a certain extent showed that the entry of light led to the development of the axial length of the right eye.

In summary, our study described a rare case of congenital pupillary abnormality. The abnormal clinical findings in our case were microcoria, amblyopia, astigmatism and exotropia. The extremely small pupil (diameter < 0.5 mm) is a peculiarity of this case and should be differentiated from acorea. The early management of this disease which leads to visual impairment and deprivation amblyopia is essential. The transparent lens should be protected as much as possible during the operation, and the fundus should be examined for those who cannot undergo fundus examination before operation. All patients need to be evaluated for the risk of amblyopia and anisometropia, especially in monocular cases.

## Data Availability

The datasets used and analysed during the current study are available from the corresponding author on reasonable request.

## Abbreviations

MCOR: Microcoria
BCVA: Best-corrected visual acuity
D: Diopter
UBM: Ultrasound biomicroscopy
OCT: Optical coherence tomography
